# Husbands’ Knowledge of Breast Cancer and Their Wives’ Attitudes and Practices Related to Breast Cancer Screening in Saudi Arabia: Cross-sectional Online Survey

**DOI:** 10.2196/25404

**Published:** 2021-02-25

**Authors:** Afnan Abdulnasir Sabgul, Ameerah M N Qattan, Rubayyat Hashmi, Mohammed Khaled Al-Hanawi

**Affiliations:** 1 Academic Affairs and Training Department King Abdulaziz Hospital Makkah Saudi Arabia; 2 Department of Health Services and Hospital Administration Faculty of Economics and Administration King Abdulaziz University Jeddah Saudi Arabia; 3 Centre for Health Research University of Southern Queensland Springfield Australia; 4 School of Business Faculty of Business, Education, Law and Arts University of Southern Queensland Toowoomba Australia

**Keywords:** attitude, breast cancer, husbands, knowledge, Saudi Arabia, screening

## Abstract

**Background:**

Despite Saudi Arabia’s free and well-established cancer care program, breast cancer incidence and mortality are rising. Husbands’ knowledge, and wives’ attitudes and practices related to breast cancer screening are not well understood in Saudi Arabia.

**Objective:**

The aim of this study was to investigate husbands’ knowledge, and wives’ attitudes and practices related to breast cancer screening in Saudi Arabia.

**Methods:**

This cross-sectional study collected data from 403 husbands in the holy city of Makkah through an online self-reported questionnaire over a period of 2 months, from May 6 to July 7, 2020. Tabulation, bivariate, and multiple regression analyses were the major tools used for data analysis. Multivariate logistic regressions were used to examine the association between husbands’ knowledge and wives’ behavior regarding breast cancer screening methods.

**Results:**

Husbands’ knowledge score (a 1-point increase) was significantly associated with the wives’ utilization of mammograms (adjusted odds ratio [AOR] 1.089, 95% CI 1.024-1.159) and breast self-examination (AOR 1.177, 95% CI 1.105-1.255). Husbands’ knowledge also influenced the wives’ attitudes toward learning about breast self-examination (AOR 1.138, 95% CI 1.084-1.195). There was no significant association between husbands’ knowledge and wives’ utilization of clinical breast examination. However, richer husbands showed a socioeconomic gradient concerning their wives’ utilization of clinical breast examinations (AOR 2.603, 95% CI 1.269-5.341).

**Conclusions:**

Overall, husbands’ knowledge of breast cancer influences wives’ attitudes and practices related to breast cancer screening methods in Saudi Arabia. Thus, interventions delivered to husbands might increase breast cancer awareness and survival.

## Introduction

Breast cancer is the leading cause of cancer-related deaths in women of over 100 countries, and is responsible for approximately 15% of all cancer deaths in women worldwide [1]. In the Kingdom of Saudi Arabia (KSA), breast cancer incidence increased by approximately 10-fold during the period of 1990-2016 [2]. Moreover, breast cancer at an advanced stage is more common in Saudi women compared to women from Western countries [3]. The incidence of breast cancer is also expected to show a rising trend in the next decade in the KSA due to the elderly age structure of the Saudi community [4,5]. If the current growth rate of breast cancer incidence in the KSA continues, it is expected to become the driving force in the projected 2015-2030 economic burden of US $159.44 billion for all cancers [6].

Breast cancer awareness contributes to the early detection of cancer presentation, and is thus necessary to reduce breast cancer incidence and mortality [7]. Evidence also suggests that a substantial proportion of breast cancer cases are self-detected [8]. Thus, high-risk groups need to be aware of breast cancer risk factors and risk reduction strategies to avoid being diagnosed in the late stages of cancer [9]. Knowledge about breast cancer can also promote utilization of the recommended screening methods for the timely detection of breast cancer [10,11]. However, women tend to have a limited level of knowledge about breast cancer in the KSA [12-14]. In addition, despite free access to health care services in the KSA [15], the level of breast cancer screening practices remains low [16]. Recent studies have also found marked socioeconomic gradients in breast cancer screening uptake among Saudi women [17].

Similar to several countries in the Middle East and the Arab world, the KSA has a conservative culture, and men usually manage many of the decisions, choices, and actions of women [18,19]. A sociocultural barrier exists for breast cancer screening practices, and failure to account for such barriers can undermine the success of well-established cancer care programs [20]. Although some studies have explored men’s knowledge in relation to women’s attitudes and practices related to breast cancer screening methods [18,19,21,22], these studies either did not explore the association between men’s knowledge and their wives’ practices regarding breast cancer screening or did not control for socioeconomic gradients. Thus, the degree to which men’s knowledge can support women to take actions for better protection from breast cancer in a country such as the KSA is not fully understood. Understanding such a mechanism might help in developing breast cancer awareness interventions and reduce breast cancer mortality through early detection.

The aim of this study was to investigate the potential effect of husbands’ knowledge on their wives’ attitudes and practices related to breast cancer screening in the holy city of Makkah in the Makkah region of western Saudi Arabia, located 70 kilometers inland from Jeddah. Approximately 26% of the KSA population live in the Makkah region [23]. Makkah also hosts the largest annual Muslim pilgrimage, the Hajj, and is thus regarded as a holy city among Muslims. Moreover, the city has a diverse population, with different lifestyle patterns and economic statuses. To understand the impact of husbands’ knowledge in Makkah city, we controlled for the possible effects of socioeconomic characteristics on wives’ attitudes and practices related to breast cancer screening in this study.

## Methods

### Study Setting and Sample

This cross-sectional study was carried out in Makkah city of the Makkah region in the KSA from May 6 to July 7, 2020. Given the social/physical distancing measures implemented in the country because of the COVID-19 pandemic, data were collected via an online self-reported questionnaire using a Google Drive website. The link was distributed to potential participants via social media, including Twitter and WhatsApp groups.

This study included participants who were residents of Makkah city and were male partners (ie, husbands) aged between 20 and 80 years. According to the latest KSA census, Makkah has a total of 434,248 married men (ie, husbands) [23]. The minimum sample size necessary for the study was calculated to be 384 husbands (margin of error of 5%, confidence level of 95%, 50% response distribution, based on the total 434,248 husbands in the region).

The questionnaire was developed by the authors based on several previous studies [3,12,14,19,20]. The questionnaire consisted of three main parts: sociodemographic characteristics of the participants, knowledge about breast cancer and screening methods, and knowledge of wives’ practices and attitudes related to breast cancer screening. The questionnaire was written in Arabic. It was initially drafted by two authors (AS and MA) in English and then translated to Arabic by AQ. The questionnaire was also back-translated to English by two bilingual speakers, and reviewed by academic researchers from the Department of Health Services and Hospital Administration at King Abdulaziz University to maintain the original construct and to ensure the meaning of the content.

On the first page of the online questionnaire, participants were provided with the study background, aim, and objectives. They were informed that they did not have to complete the questionnaire and were free to withdraw at any point without providing a reason. They were also informed that there would be no sensitive personal questions, and all information provided would be anonymous, confidential, and used for research purposes only. Participants aged 20 years or over living in Makkah city, who understood the content of the questionnaire, and agreed to participate in the study were instructed to start the questionnaire. Online informed consent was obtained from all participants before proceeding with the questionnaire.

### Variables

In this study, we defined four outcome variables: variables related to wives’ practices of breast self-examination (BSE) (yes, no, and unsure), clinical breast examination (CBE) (yes, no, and unsure), and mammograms (yes, no, and unsure), and a variable related to wives’ attitudes toward learning about BSE (yes, no, and unsure). The main exposure variable of this study was the husbands’ knowledge scores. Using predetermined answer keys based on medical evidence, each correctly answered knowledge question was awarded one knowledge point. Incorrectly answered questions or “I don’t know” responses were awarded zero knowledge points.

There were 20 knowledge questions in the questionnaire, including two questions on BSE (age of commencement and frequency), one question on CBE (age of commencement), two questions on mammograms (age of commencement and frequency), five questions on symptoms (changes in breast size, nipple morphology, nipple dryness, nipple discharge, and lymphadenopathy), six questions on risk factors (use of contraceptives, hormone replacement therapy, overexposure to radiation, smoking, hereditary, and old age), and four questions on protective factors (natural breastfeeding, sports/exercise practices, early conception, and balanced and healthy diet). The total knowledge score was computed by adding up the points for each of the 20 breast cancer knowledge questions, yielding a total breast cancer knowledge score between 0 and 20.

Multivariate logistic regression analyses were performed controlling for the husbands’ sociodemographic covariates. We used age, nationality, education, occupation, and household monthly income level as the sociodemographic control variables. Age was categorized as 20-29 (reference category), 30-39, 40-49, 50-59, and ≥60 years. Nationality was categorized as non-Saudi (reference category) and Saudi. Education was categorized as high school education level or less (reference category) and college/university degree or higher. Occupation was split into the categories of unemployed or retired (reference category), self-employed, government employee, and private sector employee. Household monthly income was grouped into two categories: less than 10,000 Saudi Riyal (SR) (reference category) and ≥10,000 SR (1 SR=US $0.27).

### Statistical Analyses

Tabulation, bivariate, and multiple regression analyses were the major data analytic tools used in this study. Means (SD) are used to describe the continuous variables, whereas frequency and percentages are used to describe the categorical variables. The nonparametric univariate χ^2^ goodness-of-fit test was used to assess the statistical significance of the husbands’ indicators of knowledge on breast cancer signs and preventive measures. Multivariate logistic binary regression analysis was used to assess the correlations between the husbands’ knowledge about breast cancer and their sociodemographic, educational, and economic factors with their wives’ various types of breast screening behaviors. The associations are expressed as adjusted odds ratios with 95% CIs. The SPSS version 20 software package was used to perform all statistical analyses.

### Ethical Clearance

All procedures performed in this study involving human participants were performed in accordance with the ethical standards of the institutional or national research committee and with the 1964 Helsinki Declaration and its later amendments or comparable ethical standards. This study was reviewed and approved by the King Abdulaziz University Research Ethics Committee, and was designed and performed in accordance with the ethical principles established by the university. This study also received ethical approval from the Ministry of Health of the KSA (IRB no: H-02-K-076-0320-275). Online informed consent to participate was secured from all respondents who participated in this study. The data collection procedure was anonymous and as such no personal identifying information was collected. The data were stored on the first author’s password-protected personal computer. An additional copy of the data was stored on a password-protected external drive kept by the first author to serve as a backup.

## Results

### Demographic and Socioeconomic Characteristics of the Respondents

A total of 445 participants completed the questionnaire. After excluding 42 respondents who reported living outside of Makkah city, the final sample consisted of 403 participants. Table 1 shows the diverse demographic and socioeconomic characteristics of the study participants.

The majority of participants were Saudi citizens, and the others comprised expatriate men residing in Makkah city. Of the 403 participants, over half were aged 40 or older, over three-quarters had a college/university or a postgraduate degree, and just over half worked in various governmental jobs. Over one-third of participants had a household monthly income of less than 10,000 SR.

**Table 1:** Demographic and socioeconomic characteristics (N=403).

Characteristic		Frequency, n (%)	95% CI
**Age (years)**			
	20-29	64 (15.9)	12.7-19.1
	30-39	120 (29.8)	25.3-34.2
	40-49	106 (26.3)	22.1-30.3
	50-59	62 (15.4)	12.4-18.6
	≥60	51 (12.7)	10.2-14.7
**Nationality**			
	Non-Saudi	56 (13.9)	10.7-17.1
	Saudi	347 (86.1)	82.9-89.3
**Educational level**			
	High school level or below	85 (21.1)	17.4-24.6
	College/university degree	248 (61.5)	57.3-66.2
	Postgraduate degree	70 (17.4)	14.1-20.6
**Occupation**			
	Retired/unemployed	79 (19.6)	16.4-22.6
	Self-employed	19 (4.7)	3.0-6.7
	Governmental employee	214 (53.1)	48.6-57.8
	Private sector employee	91 (22.6)	19.1-26.8
**Household monthly income (Saudi Riyal)^a^**			
	˂5000	39 (9.7)	7.2-12.4
	5000 to <7000	37 (9.2)	6.7-11.9
	7000 to <10,000	72 (17.9)	14.6-21.1
	10,000 to <15,000	142 (35.2)	31.3-39.5
	15,000 to <20,000	57 (14.1)	11.2-17.4
	20,000 to <30,000	42 (10.4)	7.9-13.4
	≥30,000	14 (3.5)	2.2-5.2

^a^1 Saudi Riyal=US $0.27.

### Husbands’ Knowledge and Awareness of Female Breast Cancer

Table 2 shows the husbands’ knowledge and awareness of breast cancer screening methods, including BSE, CBE, and mammograms.

In general, the basic knowledge and awareness of female BSE, CBE, and mammograms was low among the husbands in Makkah city. The majority of the husbands had heard about female BSE; however, when asked about the stage at which women should start BSE, just over a quarter of the respondents correctly inferred that they should start from the age of 20. The same proportion also correctly suggested that monthly BSE is recommended for women. In contrast, over half of the respondents had not heard about CBE, and only about 35% of the husbands correctly answered that the recommended time of CBE is from the age of 40 or when women have symptoms. Furthermore, approximately 60% of the husbands had heard about mammograms. However, less than half of the respondents correctly inferred that the recommended starting age of mammograms is from the age of 40 or higher. Similarly, only about a third of the husbands correctly suggested that the recommended frequency of mammograms for women is every 1-2 years.

The husbands’ poor knowledge of breast cancer screening was also reflected in their wives’ attitudes and practices related to these behaviors. As shown in Table 3, according to the husbands, only approximately 20% of the wives regularly self-examined their breasts or had ever had a CBE. Similarly, less than 20% of the wives had ever had a mammogram. However, over half of the husbands stated that their wives were willing to learn more about BSE.

The results related to the husbands’ knowledge of female breast cancer symptoms, risk factors, and preventive measures are shown in Table 4.

**Table 2:** Husbands’ knowledge and awareness of breast self-exams (BSE), clinical breast exams (CBE), and mammograms (N=403).

Question				Frequency, n (%)		95% CI
**BSE**						
	**Have you ever heard of female BSE?**					
		No	107 (26.6)		22.8-31.0	
		Yes	296 (73.4)		69.0-77.4	
	**At what stage do you think women should start BSE?**					
		Do not know	145 (36.0)		31.5-40.4	
		At puberty	50 (12.4)		9.4-15.5	
		From the age of 20	108 (26.8)		22.6-31.0	
		From the age of 40	92 (22.8)		19.1-26.8	
		After menopause	8 (2.0)		1.0-3.0	
	**How often should women undergo BSE?**					
		Do not know	170 (42.2)		37.7-46.7	
		Weekly	26 (6.5)		4.5-8.4	
		Monthly	106 (26.3)		22.3-30.2	
		Annually	101 (25.1)		21.1-29.3	
**CBE**						
	**Have you ever heard of female CBE?**					
		No	229 (56.8)		52.1-61.8	
		Yes	174 (43.2)		38.7-47.6	
	**At what age do you think women should start CBE?**					
		Do not know	220 (54.6)		49.6-59.3	
		At puberty	12 (3.0)		1.5-4.6	
		From the age of 20 or higher	29 (7.2)		5.2-9.2	
		From the age of 40 or when there are symptoms	139 (34.5)		30.0-39.0	
		After menopause	3 (0.7)		0.0-1.7	
**Mammogram**						
	**Have you ever heard of a mammogram?**					
		No	161 (40.0)		35.2-44.4	
		Yes	242 (60.0)		55.6-64.5	
	**At what age do you think women should start having mammograms?**					
		Do not know	199 (49.4)		44.9-54.1	
		At puberty	3 (0.4)		0.0-1.7	
		From the age of 20 or higher	20 (5.0)		3.2-6.9	
		From the age of 40 or higher	169 (41.9)		37.5-46.2	
		After menopause	12 (3.0)		1.7-4.2	
	**How often should women undergo a mammogram?**					
		Do not know	210 (52.1)		47.4-56.5	
		Monthly	12 (3.0)		1.7-4.5	
		Every 1-2 years	145 (36.0)		31.5-40.7	
		Every 4 years	36 (8.9)		6.5-11.7	

**Table 3:** Husbands’ knowledge of their wives’ practices and attitudes related to breast cancer screening (N=403).

Outcome variables		Frequency, n (%)	95% CI
**Has your wife ever had a mammogram?**			
	No	334 (82.9)	79.4-86.4
	Yes	69 (17.1)	14.1-20.6
**Has your wife ever had a clinical breast exam?**			
	No	322 (79.9)	75.9-83.4
	Yes	81 (20.1)	16.6-23.6
**Does your wife self-examine her own breasts regularly?**			
	No	328 (81.4)	77.9-84.9
	Yes	75 (18.6)	15.1-22.1
**Do you think your wife is willing to learn more about breast self-examination?**			
	No	180 (44.7)	40-49.6
	Yes	223 (55.3)	51.1-59.8

**Table 4:** Husbands’ knowledge about female breast cancer symptoms, risk factors, and preventive measures (N=403).

Question			Incorrect answer			Correct answer		GOF^a^ *χ*^2^ (*df*=2)		*P* value
			n (%)	95% CI	n (%)		95% CI			
**Symptoms**										
		The change in women’s breast size is a sign of breast cancer	285 (70.7)	66.7-75.2	118 (29.3)		25.1-33.0	106.784	<.001	
		Lymphadenopathy is a sign/symptom of breast cancer	162 (40.4)	35.7-44.7	241 (59.6)		55.3-64.3	170.462	<.001	
		Nipple skin dryness and peeling is a sign of breast cancer	299 (74.2)	70.2-78.2	104 (25.8)		22.0-29.8	175.985	<.001	
		Nipple discharge is a sign of breast cancer	273 (67.7)	63.5-72.0	130 (32.3)		27.8-36.7	172.263	<.001	
		Changes in nipple morphology is a sign of breast cancer	268 (66.5)	61.8-71.1	135 (33.5)		29.0-38.0	167.29	<.001	
**Risk factors**										
	Use of contraceptives is a possible risk factor for breast cancer		333 (82.6)	78.9-85.9	70 (17.4)		14.4-20.8	150.02	<.001	
	Hormonal replacement therapy may predispose women to breast cancer		338 (83.9)	80.1-87.1	65 (16.1)		13.2-19.6	275.275	<.001	
	Overexposure to radiation may predispose women to breast cancer		160 (39.7)	35.2-44.7	243 (60.3)		55.6-64.5	209.037	<.001	
	Cigarette smoking is a risk factor for breast cancer among women		144 (35.7)	31.0-40.2	259 (64.3)		60.3-68.7	195.042	<.001	
	Women may develop breast cancer due to hereditary factors		123 (30.5)	26.1-35.0	280 (69.5)		65.3-73.9	260.164	<.001	
	Older age is a factor that enhances the chance of developing breast cancer among women		259 (64.3)	59.6-68.7	144 (35.7)		31.5-40.0	59.201	<.001	
**Protective factors**										
	Natural breastfeeding may prevent against developing breast cancer among women		103 (25.6)	21.8-29.8	300 (74.4)		70.5-78.2	326.298	<.001	
	Regular sports/exercise may protect against breast cancer		128 (31.8)	27.4-36.3	275 (68.2)		63.8-72.2	258.169	<.001	
	Early conception before the age of 30 years may reduce the chance of getting cancer among women		325 (80.6)	77.2-84.6	78 (19.4)		15.4-22.8	258.854	<.001	
	A balanced and healthy diet may reduce the chance of developing breast cancer among women		123 (30.5)	26.3-35.7	280 (69.5)		65.3-73.7	277.97	<.001	

^a^GOF: goodness of fit.

The most frequent symptom of breast cancer that had been correctly identified was the sign of lymphadenopathy, followed by changes in nipple morphology, nipple discharge, changes in breast size, and nipple skin dryness. Similarly, the most frequent risk factor of breast cancer mentioned by husbands was hereditary factors, followed by cigarette smoking, overexposure to radiation, older age, use of contraceptives, and hormonal replacement therapy. In addition, the most frequent protective factor of breast cancer known to husbands was natural breastfeeding, followed by a balanced and healthy diet, regular exercise, and early conception before the age of 30.

The knowledge score of the husbands was calculated from the scores of the 20 knowledge questions shown in Tables 2 and 4. The score ranged from 0 to 20, with a mean knowledge score of 8.31 points (SD 4.91). Those with a lower knowledge score were regarded as having poorer knowledge of breast cancer compared to those with a higher knowledge score. The knowledge score was then used to investigate any effects of the husbands’ knowledge on their wives’ attitudes and practices related to breast cancer screening. The details of the regression analyses are discussed in the following section.

### Regression Analyses

Table 5 shows the results of the multivariate logistics regression analyses used to assess the adjusted association of husbands’ sociodemographic characteristics and knowledge about breast cancer with their wives’ attitudes and practices related to breast cancer screening. Husband age of 40-49, 50-59, and ≥60 years was significantly associated with their wives’ practices of mammogram screening. However, only age of 40-49 years was significantly associated with their wives’ practices of CBE. Similarly, a husband age of 30-39 and 40-49 years was associated with their wives’ practices of BSE. Only older husbands aged 60 years or more had significantly lower odds in terms of their wives’ willingness to learn more about BSE.

The results also revealed that husbands who were employed in the private sector had more than a 4-times higher odds that their wives had performed mammography screening than those who were retired or unemployed. Similarly, the husbands with higher household monthly income levels (≥10,000 SR) had approximately 2-times higher odds that their wife had undergone a mammogram and had more than 2-times higher odds that their wives had undergone a CBE. Additionally, the husbands’ high knowledge score was significantly associated with their wives’ utilization of mammograms and BSE, as well as their wives’ willingness to learn more about BSE. However, no association between husbands’ knowledge scores and wives’ utilization of CBE screening was found (Table 5).

**Table 5:** Multivariate logistic regression analyses of the effects of husbands’ knowledge on their wives’ attitudes and practices related to breast cancer screening.

Variables		Wife underwent a mammogram, AOR^a^ (95% CI)	Wife underwent a CBE^b^, AOR (95% CI)	Wife underwent a BSE^c^, AOR (95% CI)	Wife willing to learn more about BSE, AOR (95% CI)
**Age (years)**					
	30-39	2.084 (0.638-6.812)	2.080 (0.781-5.54)	4.595 (1.482-14.246)***	1.457 (0.761-2.788)
	40-49	4.332 (1.372-13.675)**	3.207 (1.22-8.425)**	4.363 (1.352-14.079)**	1.691 (0.862-3.318)
	50-59	5.34 (1.423-20.10)**	2.764 (0.897-8.515)	2.852 (0.722-11.263)	1.188 (0.522-2.705)
	≥60	6.54 (1.325-32.56)**	1.880 (0.419-8.432)	2.414 (0.473-12.329)	0.220 (0.069-0.698)**
Saudi nationality		1.698 (0.639-4.513)	1.305 (0.495-3.437)	2.030 (0.726-5.677)	2.091 (0.954-4.584)
College/university degree or higher		0.772 (0.355-1.67)	0.665 (0.323-1.381)	1.557 (0.641-3.780)	0.549 (0.296-1.018)
**Occupation**					
	Self-employed	2.109 (0.398-11.183)	0.327 (0.039-3.588)	1.101 (0.177-5.639)	0.599 (0.167-2.15)
	Government employee	0.646 (0.211-1.980)	0.997 (0.344-3.588)	0.387 (0.122-1.231)	0.644 (0.271-1.527)
	Private sector employee	4.735 (1.319-16.990)**	2.677 (0.789-9.084)	1.394 (0.401-4.844)	1.097 (0.409-2.939)
Household monthly income ≥10,000 SR^d^		1.965 (0.925-4.174)	2.603 (1.269-5.341)***	1.222 (0.602-2.479)	0.710 (0.411-1.228)
Knowledge score		1.089 (1.024-1.159)***	1.045 (0.989-1.108)	1.177 (1.105-1.255)***	1.138 (1.084-1.195)***

^a^AOR: adjusted odds ratio.

^b^CBE: clinical breast examination.

^c^BSE: breast self- examination.

^d^SR: Saudi Riyal (1 SR=US $0.27).

*P<.10, **P<.05, and ***P<.01.

## Discussion

### Principal Findings

Although previous studies have addressed the knowledge and attitude of breast cancer screening among Saudi women, this study illustrates the impact of husbands’ knowledge on their wives’ breast cancer screening behavior in Makkah city of the KSA using an online cross-sectional survey. The findings revealed that husbands’ knowledge has an important impact on their wives’ attitudes and practices related to the utilization of breast cancer screening methods. This study also showed socioeconomic gradients concerning wives’ utilization of mammogram and CBE.

Breast cancer survival is closely associated with cancer awareness [7,24]. Because of the conservative culture in the KSA, many women refrain from seeking medical advice until breast cancer presents at an advanced stage [25]. Moreover, some women cannot have any screening of their breasts without first obtaining approval from their male guardians [20]. Thus, men have an important role in supporting and encouraging women to obtain early diagnosis and treatment. However, the magnitude of the influence that husbands’ knowledge of breast cancer has on their wives’ screening behavior was unclear in earlier studies. Our findings have strengths that might be relevant in addressing the information needs of breast cancer patients [26] or implementing risk-stratified breast cancer prevention strategies [27].

One of the major findings of this study was that the husbands’ knowledge was significantly associated with their wives’ utilization of mammograms and BSE. This study also found that a 1-point increase in the husbands’ knowledge scores on breast cancer increased the odds of mammogram utilization by approximately 1.1 times and increased the odds of BSE utilization by approximately 1.2 times. In addition, a 1-point increase in the husbands’ knowledge scores on breast cancer increased the odds of their wives’ being willing to learn about BSE by approximately 1.1 times. If considered cumulatively, our findings suggest that the husbands’ knowledge substantially increased the probability of their wives’ participation in mammogram and BSE screening methods. Similar findings were also reported in another study [19].

Most previous studies have not controlled for all socioeconomic factors when investigating husbands’ knowledge about breast cancer [18-22]. However, several other studies found socioeconomic gradients in breast cancer incidence, mortality, and screening uptake behavior [17,28,29]. In our study, the findings of socioeconomic gradients were mixed, showing limited socioeconomic gradients in terms of mammogram (private sector employee) and CBE (income level) uptake. However, this study also found no association between education, nationality, and screening practices. This could be due to our small sample size. Despite the majority of our sample participants being highly educated, the level of breast cancer knowledge among the husbands in Makkah city was not satisfactory. Similar findings of men’s poor knowledge about breast cancer were also reported in the Jeddah [21,22], Hail [3], and Asir [14,19] regions of Saudi Arabia, as well as in other countries such as Jordan [18] and Ghana [30].

In line with existing evidence [19], our study also found a significant association between the age of the husbands and their wives’ utilization of mammograms, CBEs, and BSE, as well as their wives’ attitudes toward learning about BSE. We found that only a husband age of 40-49 years had a significant relationship with CBE uptake, whereas almost all ages (40-49, 50-59, and 60 years or older) showed a significant association with mammogram uptake. BSE was usually performed by the wives of younger husbands (30-39 and 40-49 years), whereas the wives of elderly husbands (60 years or older) were less willing to learn about BSE. Our findings suggest that the age distribution of the husbands had a significant impact on their wives’ utilization of screening methods and attitudes toward breast cancer.

In the KSA, a large number of breast cancer cases are discovered at late stages, which leads to a lower rate of recovery [31]. Despite free health services and free screening facilities, there is almost no use of mammograms in the KSA [15,16]. Thus, increasing the awareness about early detection tools for breast cancer is vitally important. Recent studies have found that breast cancer awareness interventions (eg, public health campaigns and educational programs) are effective to increase the uptake of BSE and mammogram behaviors, and to increase the probability of breast cancer screening attendance [32]. Studies have also found that target-based interventions can increase breast cancer awareness [9]. Our study findings indicate that there is a lot of work that needs to be done to improve cancer survival rates and to lower incidence rates. It is strongly recommended to empower women, raise cancer awareness, and remove barriers to fight breast cancer.

### Limitations

The findings of this study should be interpreted in light of some limitations. This research concerned the Makkah region and urban settings only, where the majority of the men were well educated. In addition, the study design was cross-sectional in nature with the possibility of recall and self-reporting biases. Finally, the descriptive analysis cannot be generalized to all husbands in the KSA, as study participants were recruited through a convenience sampling method. However, care was taken to recruit participants that preserve the sociodemographic distribution of the KSA and, as already discussed, our descriptive analysis did not contradict the findings of past studies.

### Conclusions

This study found evidence that husbands’ knowledge about breast cancer has an important role in encouraging the breast cancer screening behaviors of their wives. Furthermore, the study findings documented limited knowledge about breast cancer among Makkah residents. Moreover, the wives of elderly husbands, according to their husbands’ beliefs, are less willing to learn about BSE, whereas younger husbands’ wives had higher odds of performing BSE. We also found some limited socioeconomic gradients regarding the utilization of mammograms and CBEs. These findings have important implications for conservative cultures where men play a crucial role in women’s decisions and actions. Policies could be directed to raise breast cancer awareness, and interventions could be devised such that high-risk groups (eg, elderly or low socioeconomic status background) adhere to the recommended cancer screening protocol. Efforts should be made to empower women so that barriers to seeking medical help are alleviated. Future research is needed to evaluate whether implementing breast cancer awareness interventions increases cancer survival.

## Abbreviations

BSE: breast self-examination
CBE: clinical breast examination
KSA: Kingdom of Saudi Arabia
SR: Saudi Riyal
